# A Comparison of the Properties of Mesenchymal Stem Cells Derived from Different Synovial Sources: A Systematic Review

**DOI:** 10.3390/ijms27125582

**Published:** 2026-06-20

**Authors:** Moiz Ahmad, Jazvir Singh Kapoor, Wilegoda A. D. C. S. Wilegoda, Max Liu, Wasim Khan

**Affiliations:** 1 School of Clinical Medicine, University of Cambridge, Cambridge CB2 0SP, UK; ma2039@cam.ac.uk (M.A.); jk879@cam.ac.uk (J.S.K.); ww405@cam.ac.uk (W.A.D.C.S.W.); ml2070@cam.ac.uk (M.L.); 2 Division of Trauma & Orthopaedic Surgery, Addenbrooke’s Hospital, University of Cambridge, Cambridge CB2 0QQ, UK

**Keywords:** mesenchymal stem cells, synovium, synovial fluid, synovial membrane, chondrogenesis, cartilage regeneration, osteoarthritis, cellular heterogeneity

## Abstract

Mesenchymal stem cells (MSCs) are capable of self-renewal and differentiation into different cellular lineages, including adipocytes, chondrocytes, and osteocytes. This makes them strong candidates for repairing degenerative joint conditions such as osteoarthritis, in which native cartilage lacks repair capacity. The synovium is an attractive MSC source, with synovial MSCs demonstrating superior chondrogenic and proliferative potential compared to those from bone marrow or adipose tissue. The synovial joint is a heterogeneous environment, and MSCs can be isolated from the membrane, fluid, different histological subtypes of fibrous and adipose synovium, and different anatomical regions of synovium. This systematic review assesses whether MSCs from different synovial sources possess distinct properties. 2312 papers were identified, of which 10 met the inclusion and exclusion criteria and were included in the final review. Significant differences were identified in proliferation characteristics, immunophenotype and differentiation potential. Proximity to vasculature appeared to correlate with proliferation and differentiation potential, and MSCs from the synovial membrane may have superior proliferative characteristics compared to those from synovial fluid. More work is required to fully characterise these differences and understand their underlying molecular bases, but these findings may help inform the choice of MSC source for regenerative therapies.

## 1. Introduction

### 1.1. Articular Cartilage Injury

Articular cartilage is a specialised connective tissue that lines the articulating surfaces of synovial joints, providing a low-friction, load-bearing surface essential for normal joint function [1]. Unlike most tissues, articular cartilage is avascular, aneural and alymphatic, thus lacking capacity for intrinsic repair [2]. The loss of this articular cartilage characterises degenerative joint conditions like osteoarthritis. Current treatment approaches include lifestyle modification and pharmacological management, with joint replacement being an option for patients whose quality of life is significantly affected. Ultimately, these current treatments only provide symptomatic relief and do not restore the native joint tissue [3]. There is thus a large therapeutic gap for regenerative treatments which restore joint architecture.

### 1.2. Mesenchymal Stem Cells

Mesenchymal stem cells, also known as mesenchymal stromal cells (MSCs), are multipotent stromal cells capable of self-renewal and differentiation into mesodermal lineages, including chondrocytes, osteocytes and adipocytes [4]. Although first isolated from bone marrow in the 1970s [5], MSCs have since been isolated from a wide range of tissues, including adipose tissue, umbilical cord blood, dental pulp and synovial tissue [6]. The International Society for Cell and Gene Therapy (ISCT) defines MSCs as being adherent to plastic under standard culture conditions, expressing CD73, CD90, and CD105, while lacking CD45, CD34, CD14 or CD11b, CD79α or CD19 and HLA-DR, and capable of trilineage differentiation in vitro [7]. Beyond their ability to differentiate into different cellular lineages, MSCs can exert therapeutic effects through immunomodulation and paracrine signalling, influencing the local tissue microenvironment to promote repair [8,9]. These properties have made MSCs a leading candidate for musculoskeletal regeneration, and there are a growing number of MSC-based therapies advancing into clinical trials [10,11].

However, MSCs are not a homogeneous population. It is now well-established in the literature that MSCs from different tissue sources have significant phenotypic and functional heterogeneity [12]. This heterogeneity has direct consequences for clinical translation, with the source of MSCs potentially influencing their therapeutic utility [13].

### 1.3. Synovial Mesenchymal Stem Cells

Among adult tissue sources, the synovial joint has emerged as a particularly attractive MSC source for regenerative applications. De Bari and colleagues first demonstrated that multipotent MSCs could be isolated from adult human synovial membrane, retaining their differentiation capacity even after extensive in vitro expansion [14]. In a landmark comparative study by Sakaguchi and colleagues, MSCs from human synovial membrane showed the highest proliferative and chondrogenic capacities compared with MSCs from bone marrow, periosteum, adipose tissue, and skeletal muscle [15].

However, the synovial joint is a complex environment consisting of several different populations of MSCs. The synovial membrane (SM) comprises a thin intimal lining of macrophage-like (type A) and fibroblast-like (type B) synoviocytes, overlying a subintimal stroma varying from predominantly fibrous (FS) to adipose (AS) depending on location [16]. MSCs can also be isolated from synovial fluid (SF), which is believed to originate from the synovial membrane and mobilised into the fluid following injury or in the context of degenerative joint disease [17,18]. Further variation exists within the synovial membrane, where MSCs can be isolated from different anatomical regions within the joint or different surface, stromal and perivascular fractions, each with its own distinct microenvironmental niches that may give rise to MSC populations with distinct properties.

Given this heterogeneity, it is plausible that MSCs derived from these different synovial sources will differ phenotypically and functionally, and this has direct implications for choosing the source of MSCs for any cell-based musculoskeletal therapy. Whilst individual primary studies have compared MSCs from different synovial sources, to our knowledge, no systematic review has synthesised the current evidence base across the full range of synovial source comparisons.

### 1.4. Aims of This Review

This systematic review will compare the properties of human MSCs derived from different synovial source tissues, including synovial fluid and synovial membrane, fibrous and adipose synovium and regional anatomical variation within the membrane. We will synthesise evidence about the functional properties of these MSCs, like proliferation and clonogenicity, differentiation potential, and immunophenotype. By collecting and critically appraising the current literature, we aim to inform appropriate source selection for synovial MSC-based regenerative therapies and identify key evidence gaps to guide future translational research.

## 2. Materials and Methods

### 2.1. Search Strategy

A systematic review of the literature was conducted and reported in accordance with the Preferred Reporting Items for Systematic Reviews and Meta-analyses (PRISMA) guidelines [19]. The review protocol was prospectively registered on the International Prospective Register of Systematic Reviews (PROSPERO) database (registration number: CRD420261280555).

A structured search of the literature was performed across four electronic databases: PubMed, Embase, Scopus and Web of Science. Searches were executed on 20 December 2025, with no restrictions on publication date. Each search combined three concept blocks: (1) synovium and related terms; (2) mesenchymal stem cells; and (3) characterisation, proliferation, differentiation or expansion, with these concepts being joined with the Boolean operator “AND”. Medical Subject Headings (MeSH) and Emtree terms were used where available, together with free-text terms and truncation operators (“*”, “?”) to maximise sensitivity. The full database-specific search strings are found below:

- PubMed: (“Synovial Membrane”[Mesh] OR synovium) AND (“Mesenchymal Stem Cells”[Mesh] OR “Stem Cells”[Mesh] OR mesenchymal stem cells) AND (“Cell Differentiation”[Mesh] OR “Cell Proliferation”[Mesh] OR “Tissue Culture Techniques”[Mesh] OR “Tissue Engineering”[Mesh] OR “Tissue and Organ Harvesting”[Mesh] OR character*)—567 records.
- Embase: (exp mesenchymal stem cell/ OR exp mesenchymal stroma cell/ OR exp stem cell/) AND (exp synovium/ OR exp synovial fluid/) AND (exp cell proliferation/ OR exp cell expansion/ OR exp cell differentiation/)—648 records.
- Scopus: (synovi*) AND (mesenchymal stem cell* OR mesenchymal stromal cell* OR stem cell*) AND (character* OR proliferation OR differentiation OR expansion)—434 records.
- Web of Science: (TI=(synovi*) OR AB=(synovi*)) AND (TI=(“mesenchymal stem cell*” OR “mesenchymal stromal cell*” OR MSC*) OR AB=(“mesenchymal stem cell*” OR “mesenchymal stromal cell*” OR MSC*)) AND (TI=(characteri?ation OR proliferation OR differentiation OR expansion) OR AB=(characteri?ation OR proliferation OR differentiation OR expansion))—662 records.

**Figure 1:**
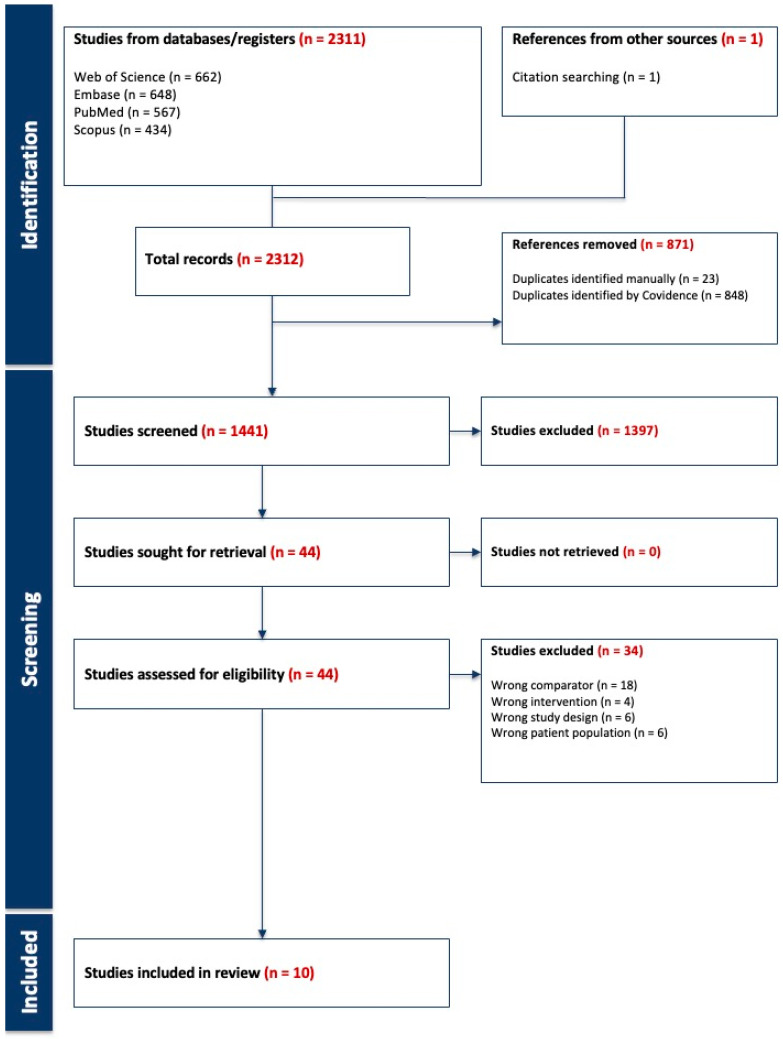
PRISMA flow diagram of study selection.

A total of 2311 records were identified across the four databases, with one study being included after citation searching, resulting in a total of 2312 records. Records were imported into Covidence (Veritas Health Innovation, Melbourne, Australia) for deduplication, after which 871 duplicates were removed, leaving 1441 studies for screening. Screening was conducted in two sequential phases. In the first phase, titles and abstracts were screened independently by at least two independent reviewers (MA and JK, or WW or ML) against the pre-specified eligibility criteria. In Phase 2, the full texts of records retained from Phase 1 were independently reassessed for eligibility by at least two independent reviewers. Inter-rater agreement for screening was moderate (Cohen’s κ = 0.49 for title/abstract and 0.59 for full-text screening). Any conflicts that arose from this screening were resolved by discussion between the authors. The study selection process is summarised in the PRISMA flow diagram in Figure 1.

### 2.2. Inclusion Criteria

1. Primary in vitro research studies performed using human tissue.
2. Studies in which MSCs were isolated from at least one defined synovial source (e.g., synovial membrane, synovial fluid, histological subtype of synovium (FS or AS), or a defined anatomical region of the synovium).
3. Studies in which synovial MSCs from two or more sources were derived from the same donor and compared in a donor-matched manner.
4. Studies reporting at least one functional outcome measure of MSCs—namely, proliferation rate, colony-forming efficiency, and/or trilineage (chondrogenic, osteogenic, adipogenic) differentiation potential.
5. Full-text articles published in the English language.

### 2.3. Exclusion Criteria

1. Studies that did not isolate MSCs (e.g., studies characterising chondrocytes, fibroblast-like synoviocytes or other non-MSC populations).
2. Non-human studies (in vitro or in vivo).
3. Studies deriving MSCs only from non-synovial sources (e.g., bone marrow, subcutaneous adipose tissue).
4. Studies isolating MSCs from only a single synovial source, without comparison to a second synovial source from the same joint.
5. Studies in which the synovial MSC populations being compared were not donor-matched.
6. Studies that did not characterise any functional property of the MSCs (e.g., proliferation rate, colony-forming efficiency, or trilineage differentiation potential).
7. Studies that did not specify the anatomical location within the synovium from which the MSCs were derived.
8. Non-primary research, including narrative reviews, systematic reviews, meta-analyses, editorials, commentaries, letters, conference abstracts and case reports.
9. Studies not available in the English language.

### 2.4. Data Extraction

Data from the studies retained after full-text screening were extracted using a pre-piloted Microsoft Excel spreadsheet. Extraction was performed by two reviewers, with disagreements resolved by discussion. The following information was recorded for each study: bibliographic details (author details, year of publication, journal, DOI); study characteristics (number of donors, age, sex, disease status, joint sampled); methodological parameters (synovial source(s) compared, tissue harvest method and the passage number(s) at which outcomes were assessed); and outcome data (proliferation, colony-forming efficiency, surface antigen expression, trilineage differentiation potential, and any additional comparisons reported). 

### 2.5. Quality Assessment

The methodological quality and risk-of-bias of each included study were assessed using a modified version of the OHAT Risk of Bias Rating Tool for Human and Animal Studies, developed by the Office of Health Assessment and Translation (OHAT) of the National Toxicology Program [20]. Each study was independently rated by two reviewers across the following domains: randomisation of administered dose or exposure level; allocation concealment; appropriate participant selection for comparison; accounting for important confounding and/or modifying variables; consistency of experimental conditions across study groups; blinding of research personnel; incomplete outcome data; confidence in exposure characterisation; confidence in outcome assessment (including assessor blinding); complete reporting of measured outcomes; and other potential threats to internal validity. Ratings for each domain were recorded as definitely low (“++”), probably low (“+”), probably high (“−”), definitely high (“−−”) risk of bias, or not applicable (“N/A”). Any discrepancies were resolved by discussion and consensus between the reviewers. 

### 2.6. Data Synthesis

Owing to substantial heterogeneity between included studies regarding donor characteristics, sources of MSCs and outcomes assessed, a quantitative meta-analysis was not considered appropriate. A narrative synthesis was therefore performed. Studies were grouped according to the sources of synovial MSCs compared, with three groups of comparisons arising: (i) synovial fluid versus synovial membrane; (ii) fibrous versus adipose synovium; and (iii) intra-articular regional variation within the synovium of the same joint. Within each domain, findings were further organised by outcome category (cellular expansion and clonogenicity, immunophenotype, and differentiation potential).

## 3. Results

### 3.1. Characteristics of Included Studies

Ten studies met the inclusion criteria, all using human tissues and performing comparisons of donor-matched samples. These studies addressed three domains of comparison. Three studies compared MSCs from synovial fluid (SF-MSCs) and synovial membrane (SM-MSCs) [21,22,23], four compared MSCs from fibrous synovium (FS-MSCs) and adipose synovium (AS-MSCs) [24,25,26,27], and three studies assessed synovial MSCs from different regions of the joint [28,29,30]. Sample sizes ranged from 8 to 40. The knee was the most sampled joint (eight studies), whilst one study assessed MSCs from the ankle and one from the hip. Disease status of donor joints ranged from healthy to osteoarthritis and chronic lateral ankle instability.

Table 1 provides a summary of the general characteristics of the included studies.

### 3.2. Synovial Fluid Versus Synovial Membrane

#### 3.2.1. Cellular Expansion and Clonogenicity

All three studies which compared MSCs from SF and SM assessed their proliferative characteristics. Findings were broadly consistent with the superiority, or at least equivalence, of SM-MSCs compared to those from SF, although the magnitude of the advantage varied with the specific metric and degree of disease.

SM-MSCs were shown to have a higher nucleated cell yield, along with greater CFE compared to SF-MSCs [21]. The initial MSC proportion was also seen to be greater for SM-MSCs compared to those from SF, although this difference in MSC availability was not present across all passages [23].

SM-MSCs displayed superior, or at least comparable, proliferation rates and had shorter population doubling times than those from SF. One study found that SM-MSCs consistently had shorter population doubling times compared to those from SF across all passages assessed, resulting in a greater yield by day 70 of expansion [21]. The same study demonstrated that SM-MSCs had a significantly higher proportion of cells in the S-phase of the cell cycle than SF-MSCs, potentially explaining the observed proliferative differences. These findings were corroborated by another study, which showed that SM-MSCs had more cumulative population doublings by P8 compared to SF-MSCs (13 vs. 11), although no formal significance of this difference was tested [22]. The same study found that SM-MSCs had shorter population doubling times across the first three passages compared to SF-MSCs, although again, no formal significance for this difference was tested.

However, in contrast to these findings, one study found no significant difference in the proliferation rates between the two MSC sources from the time of initial isolation to passage two [23].

Disease status was seen to differentially modulate the cellular availability of SM- and SF-MSCs. In one study, the Kellgren–Lawrence (KL) score of patients with knee osteoarthritis negatively correlated with SF-MSC proportion at harvest [23]. However, no correlation between the KL score and the SM-MSC proportion was found.

These findings are summarised in Table 2.

#### 3.2.2. Immunophenotype

All three studies assessed the immunophenotype of SF- and SM-MSCs. The ISCT minimum criteria for MSC identification require positive expression of CD105, CD73, and CD90 with a negative expression of CD45, CD34, CD14 or CD11b, CD79α or CD19 and HLA-DR [7]. Notably, no study assessed a complete panel of these markers but instead assessed a subset.

However, of the assessed ISCT markers, expression patterns were broadly comparable between the two MSC sources, with markers tested showing positive or negative expression as expected. The most notable source-dependent differences in immunophenotype concerned CD105 and CD34. CD105 expression was slightly lower and more variable in SF-MSCs compared to SM. One study found that CD105 was expressed at a range of 77–87% amongst SF-MSCs, compared to a maximum of 90.2% in SM-MSCs, although this difference did not reach significance [22]. Another study, however, did show the lower expression of CD105 in SF-MSCs to be statistically significant (99.00 ± 0.72% vs. 99.80 ± 0.09%; *p* = 0.043) [21], although the authors did not consider this biologically significant.

The same study found that residual CD34 positivity was significantly higher for SM-MSCs compared to SF (23.70 ± 21.50% vs. 2.65 ± 3.38%; *p* = 0.043). However, contrasting this, another study found that residual CD34 expression was greater in SF-MSCs, although no formal significance was established [23].

One study performed a more comprehensive assessment of 242 markers beyond those in the ISCT criteria, identifying a cluster expressed to a significantly greater extent in SM-MSCs compared to SF [21]. These markers included CD106 (VCAM-1: 9.59 ± 6.39% vs. 3.42 ± 1.78%), CD107a (21.10 ± 11.50% vs. 5.44 ± 3.54%), CD121a (10.60 ± 8.06% vs. 1.62 ± 0.86%), CD140b/PDGFRβ (99.10 ± 1.07% vs. 77.90 ± 24.70%), CD141 (63.70 ± 24.00% vs. 28.80 ± 32.30%), and CD143 (75.40 ± 9.23% vs. 30.00 ± 28.90%; all SM vs. SF; all *p* < 0.05).

An interesting link between the expression of some of these markers and proliferation rates was also observed in this study. Across both cell sources, CD140a and CD140b expression was negatively correlated with population doubling time (see Table 2).

These findings are summarised in Table 3.

#### 3.2.3. Differentiation Potential

Two studies compared the differentiation potential of SF- and SM-MSCs [21,23]. In contrast to the apparent proliferative superiority of SM-MSCs, differentiation properties were broadly equivalent across these MSC sources.

Both studies performed in vitro assessments of functional differentiation. This involved culturing MSCs within media designed to induce osteogenesis, chondrogenesis and adipogenesis. The resulting cells were then stained for the products of these respective processes, for example, safranin O for glycosaminoglycans produced by chondrogenesis, Oil Red O for lipid droplets from adipogenesis and alizarin red for calcium mineralisation from osteogenesis. Qualitative or quantitative assessments of these products can then be performed to compare differentiation potential. Expression of various genes involved in these processes can also be compared between MSC sources as a marker of differentiation potential.

Both studies confirmed trilineage differentiation potential but did not make overt comparisons between MSC sources [21,23]. However, one of these studies supplemented this by performing an in vivo assessment of differentiation potential, creating an osteochondral defect in rats and subsequently transplanting SF- or SM-MSCs. Repair was then semi-quantitatively scored using the International Cartilage Repair Score (ICRS). Again, no significant differences between SM- and SF-MSCs were noted [21].

These findings are summarised in Table 4.

### 3.3. Fibrous Versus Adipose Synovium

#### 3.3.1. Cellular Expansion and Clonogenicity

Four studies compared the proliferative characteristics of FS- and AS-MSCs [24,25,26,27]. One of these studies also assessed the proliferative characteristics of MSCs from subcutaneous fat, which served as a non-synovial MSC control for the FS- and AS-MSC comparison of interest [24]. In contrast to the SF–SM comparison domain, evidence for consistent differences in proliferation between FS- and AS-MSCs is weaker, with findings varying with culture conditions, age and species.

Nucleated cell yields between FS- and AS-MSCs were broadly equivalent across two studies [24,26]. However, one of these studies found that the initial MSC proportion was significantly higher in FS than AS (4.54 ± 4.05% vs. 2.58 ± 2.36%; *p* < 0.05) [26].

Evidence for consistent differences in CFE was likewise weak. Two studies, one using MSCs from the knee and one from the ankle joint, found comparable CFE between FS- and AS-MSCs [24,27]. However, another study using the knee joint found that CFE was significantly greater for FS-MSCs [25].

Proliferation rates were similarly variable across studies, with some evidence suggesting that culture conditions or donor age could differentially alter these properties. One study found no significant differences in proliferation rates between FS- and AS-MSCs, yielding a similar number of cells across all eight passages assessed [27]. Another study found that AS-MSCs expanded more than those from FS, but only when cells were isolated using a collagenase method and not an explant culture [26], suggesting an interaction between tissue source and isolation method.

Donor age and culture conditions were also seen to potentially differentially modulate the proliferative properties of FS- and AS-MSCs. One study found that in mixed-population cultures, FS-MSCs had a significantly higher fold increase in cell number compared to those from AS (approximately 100-fold vs. approximately 60-fold; *p* < 0.05) [24]. However, this proliferative difference was absent in elderly donors. Moreover, when single-clonal-derived populations were assessed, this difference in proliferation between FS- and AS-MSCs was also no longer present, suggesting that any apparent differences in proliferation rates were due to heterogeneous cellular populations rather than intrinsic differences in MSC populations.

These findings are summarised in Table 5.

#### 3.3.2. Immunophenotype

Three studies compared the immunophenotype of FS- and AS-MSCs [24,26,27]. All found a highly conserved profile between the two MSC sources, with cells being positive and negative for ISCT markers as expected. The exception is that one study did identify CD105 expression across both FS- and AS-MSCs from the ankle as being only 6.3% and 8.5%, respectively, well below the positive expression seen in other studies [27]. Indeed, this falls far below the ISCT-defined threshold of ≥95% expression [7]. The implications of this for the classification of these populations of cells are considered in Section 4.3.

These findings are summarised in Table 6.

#### 3.3.3. Differentiation Potential

Three studies confirmed trilineage differentiation potential for MSCs from both sources, with no study reporting a failure of either source to differentiate [24,26,27]. Across two studies, differentiation potential was uniformly equivalent, with no significant differences in chondrogenic pellet weight, glycosaminoglycan content, or osteogenic/adipogenic colony ratios arising [24,26].

One study did, however, find significant quantitative differences in adipogenic differentiation potential between FS and AS [27]. AS-MSCs had a significantly higher Oil Red O-positive colony rate following adipogenic induction compared to FS (65% vs. 30%, *p* < 0.05). This was also reflected at the transcriptomic level. The expression of adipsin was seen to be significantly higher in AS-MSCs compared to those from FS (approximately 2.5× fold, *p* < 0.05). Whilst the same study showed the expression of PPAR-γ and LPL to also be higher than FS (4× and 5× differences, respectively), these differences did not reach statistical significance.

These findings are summarised in Table 7.

### 3.4. Intra-Articular Variation

Three studies assessed within-joint regional variation in MSC properties [28,29,30]. Across studies, the consistent finding was that the properties of synovial MSCs differ according to the precise harvest site within the joint. Table 8, Table 9 and Table 10 summarise the main findings from these studies regarding differences in clonogenicity and proliferation, immunophenotype and differentiation potential of the MSC sources assessed, respectively.

#### 3.4.1. Clonogenicity and Proliferation

Colony-forming efficiency varied by as much as two- to five-fold depending on the harvest site within the synovial membrane. One study reported that CFE was highest for SM-MSCs derived from the medial outer capsule and lowest in the medial inner region of the knee [30], varying approximately fourfold from ~40–50 colonies per 1000 nucleated cells to ~10–20 colonies per 1000 nucleated cells, respectively. These differences in CFE correlated directly with local vascular density, as characterised by α-SMA^+^ and CD31^+^ vessel counts.

Significant differences in CFE were also seen in the hip [29]. This study demonstrated that MSCs from the cotyloid-fossa synovium had a 3.5-fold higher CFE (31.6 ± 23.7 vs. 9.0 ± 15.7 per 10^4^ cells; *p* < 0.01) and an 11-fold greater cell yield at P0 (*p* < 0.01) compared with MSCs from the paralabral synovium, and expanded to P14 versus P10 for paralabral-derived cells. Another study showed that MSCs from the perivascular fraction of synovial membrane had a greater proliferation rate than MSCs from surface or stromal regions (approximately 125-fold vs. approximately 50–70-fold for surface and stromal fractions; *p* < 0.01) [28].

#### 3.4.2. Immunophenotype

One study identified unique immunophenotypes of MSCs from perivascular, stromal and surface fractions of SM [28], particularly with respect to CD55 and CD271: surface CD55^+^/CD271^−^, perivascular CD55^−^/CD271^+^, and stromal cells negative for both markers. However, after in vitro expansion, the immunophenotypes of these subpopulations converged, and the differences between fractions were no longer significant. Whilst only focusing on MSCs from one particular region of the synovium (suprapatellar pouch), another study likewise found a significant decline in the expression of several markers, like CD105 and CD90, over 21 days of culture [30].

#### 3.4.3. Differentiation Potential

Differences in differentiation potential paralleled the differences in proliferation. In the hip, MSCs from the cotyloid fossa had superior colony rates for adipogenesis (64.5% vs. 45.4%; *p* < 0.05) and osteogenesis (59.8% vs. 39.7%; *p* < 0.05), and higher expression of chondrogenesis-related genes (e.g., COL2A1 19.9-fold higher; *p* < 0.01) compared to MSCs from paralabral synovium [29]. In the knee, MSCs from the perivascular fraction of synovial membrane had higher chondrogenic and calcification potential compared to other SM fractions, whilst adipogenic potential was broadly comparable across fractions [28]. However, another study found that whilst different regions of knee synovium had differing proliferative characteristics, chondrogenic potential did not differ significantly between harvest sites [30].

### 3.5. Risk of Bias Assessment

The quality of each included study was evaluated using a modified version of the OHAT (Office of Health Assessment and Translation) Risk of Bias Rating Tool for Human and Animal Studies, applying the eleven domains outlined below. Each study was independently rated on each applicable domain as definitely low risk (++), probably low risk (+), probably high risk (−), or definitely high risk (−−). Domains relating to administered dose randomisation and allocation concealment were not applicable (N/A) to this body of literature, as all included studies were observational comparisons of MSCs isolated from different synovial sources rather than experimental intervention trials.

Overall, all ten studies were judged to be of acceptable methodological quality, and all of them were considered to be at low overall risk of bias. All ten studies were rated probably high risk (−) for blinding of research personnel during the experimental workflow, as none explicitly described blinding of personnel performing experimental procedures. For confidence in outcome assessment, eight studies were also rated probably high risk (−) for the same reason. Two studies were rated probably low risk (+) on this domain because each explicitly described blinded scoring of its primary endpoint: Katagiri et al. [25] reported colony counting by three independent observers in a blind manner, and Amemiya et al. [21] described independent blinded scoring of cartilage regeneration sections by two researchers. Confidence in exposure characterisation was uniformly high, with anatomical source, isolation method, and processing workflow clearly described in all studies. Confounding was adequately managed in most studies through donor-matching of MSC sources or explicit stratification by donor characteristics, although three studies (Ferro et al. [22], Katagiri et al. [25] and Mizuno et al. [28]) raised concerns owing to limited reporting of donor demographics or a heterogeneous donor population.

Risk-of-bias ratings per domain are presented in Table 11.

## 4. Discussion

### 4.1. Summary of Evidence

This systematic review has synthesised evidence from ten donor-matched human studies addressing three domains of comparison: SF vs. SM, FS vs. AS, and intra-articular regional variation within the SM. A key conclusion is that the source of MSCs within the synovial joint has a measurable influence on properties like immunophenotype, proliferation and differentiation potential. These findings are consistent with a profound influence of the local microenvironment on the properties of synovial MSCs.

### 4.2. Synovial Fluid vs. Synovial Membrane

Across the three studies in this domain, SM-MSCs appear to have superior or at least comparable proliferation characteristics compared to those from SF. Two of the three studies reported a numerical SM-MSC proliferative advantage which appeared to be sustained, not just confined to early expansion [21,22]. However, there was heterogeneity amongst these studies, with one failing to test the significance of the higher proliferation rates of SM-MSCs observed [22]. Another study reported no significant proliferative differences between MSC sources [23]. Differentiation potential was seen to be comparable between MSC sources. Immunophenotypes were broadly similar between the two MSC types, both meeting the ISCT criteria. Whilst small differences in expression of certain markers like CD105 and CD34 were observed, these were not thought to be biologically significant or did not reach statistical significance. However, one study performed a more extensive investigation of non-ISCT markers, finding significantly higher expression of pericyte-adjacent markers in SM-MSCs like CD140b and CD106 [21].

SF-MSCs are generally considered to originate from the SM, being mobilised in response to injury [17,18]. The studies from this review support this hypothesis. Lee et al. [23] found that the SF-MSC, but not SM-MSC, proportion correlated with the severity of knee osteoarthritis. Further supporting this hypothesis is the largely similar immunophenotype and differentiation potentials of MSCs from the two sources.

The apparent proliferative superiority of SM-MSCs compared to those from SF in two studies is of note. One hypothesis to explain these proliferative differences, despite a shared origin for the two MSC sources, is that upon mobilisation from the SM, SF-MSCs may lose paracrine trophic support from the SM. This hypothesis is supported by the findings of Amemiya et al. [21], who conducted an extended immunophenotyping panel to find that SM-MSCs had a higher expression of, amongst other markers, CD140b, the receptor for PDGF-β, compared to SF-MSCs. This potential mechanism, along with other mechanisms to explain the source-dependent differences in MSC properties identified in this review, is explored in detail in Section 4.5.

Ultimately, there is limited evidence based on this matter. Individual study sample sizes limit statistical interrogation. More studies with larger sample sizes that are sufficiently powered to detect differences in proliferation rates between MSC sources are required. These studies should also seek to understand the underlying molecular basis for any proliferative differences between SF- and SM-MSCs. Such experimental approaches are explored in more detail in Section 4.6.

### 4.3. Fibrous vs. Adipose Synovium

Across the four studies comparing FS- and AS-MSCs, evidence for consistent differences in MSC properties was weak and frequently appeared to depend on isolation method, donor age and the joint sampled.

Whilst nucleated cell yields were seen to be broadly comparable across two studies [24,26], one of these studies found a significantly higher initial MSC proportion within FS- than AS-MSCs [26]. CFE findings were conflicting: comparable between the two sources in two studies [24,27] but significantly higher in FS- than AS-MSCs in another study [25]. Most studies found comparable trilineage differentiation potential and immunophenotype [24,25,26], although one study did find significantly greater adipogenic potential for AS-MSCs compared to those from FS [27].

The studies in this review highlighted interesting interactions between experimental method and apparent functional differences between the two MSC sources. Lee et al. [26] found that AS-MSCs expanded more than those from FS, although only when cells were isolated using a collagenase method—no difference in expansion was seen when an explant method was used. This suggests that any proliferative advantage is not due to intrinsic differences between MSCs but rather a property of the experimental method. On a similar note, Mochizuki et al. [24] observed FS-MSCs from young but not elderly donors having a higher fold increase compared to those from AS. However, this difference was not present when single-cell-derived clonal populations were studied, rather than mixed-population cultures. Again, this suggests that any observed differences in MSC properties might not represent intrinsic differences in MSCs but are a reflection of their environment.

That heterogeneity can arise during culture and confound results is established within the broader MSC literature [12,31,32]. Examples of studies from our review which demonstrate these processes and some of their underlying molecular bases are discussed in Section 4.5. The findings from this review thus strengthen the importance of careful interpretation of any apparent source-dependent properties of MSCs and emphasise the importance of explicit testing for clonal drift.

One observation from this domain that warrants particular attention is the low CD105 expression of FS- and AS- MSCs from the ankle described by Nakashima et al. [27]. As discussed, the expression of CD105 in these populations fell well below the minimum ISCT criteria of at least 95% expression [7]. Moreover, the expression of another canonical marker, CD90, was also seen to be below this 95% expression threshold (89.8% and 89.2% expression for FS- and AS-MSCs, respectively), albeit not to the same extent as CD105. The expression of another canonical MSC marker, CD73 [7], was also not assessed. This would suggest that these cell populations do not meet the ISCT definition of MSCs. This apparent discrepancy was not addressed by the authors, although their findings of multilineage differentiation potential of these cell populations do suggest that they do have MSC-like properties, notwithstanding that these populations did not meet the ISCT minimum surface expression.

There are several possible explanations for these discrepancies. The low expression of CD105 may reflect genuine biological differences between the ankle and the other joints, like the hip and knee. Alternatively, the low expression of CD105 may be an artefact of culturing conditions, with evidence from another study in this review providing evidence for changes in cell-surface expression of markers with prolonged culture [28].

In any case, this finding does limit the confidence of cross-study comparison but also highlights the importance of functional, rather than purely immunophenotypic, confirmation of MSC identity.

### 4.4. Intra-Articular Regional Variation

A strong finding from this review is that MSCs harvested from different anatomical regions of the synovium can have significantly different properties. Both in the hip and the knee, proliferation rates and CFE of synovial MSCs varied by as much as five-fold [28,29,30] depending on harvest site. Whilst differences in differentiation potential were also seen to match these proliferative differences in some studies [28,29], this was not a consistent finding [30].

A consistent finding is that these proliferative and differentiation advantages appeared to mirror vascular access. Nagase et al. [30] found that SM-MSC proliferation rates correlated with the local vascular density quantified by α-SMA^+^ and CD31^+^ vessel counts, with Mizuno et al. [28] corroborating this in the knee by showing that perivascular SM-MSCs had the highest proliferation rates compared to those from surface and stromal fractions. Likewise, in the hip, MSCs from the more vascular cotyloid fossa of SM had higher proliferation rates than those from the less vascular paralabral synovium [29].

Proximity to the local perivascular niche thus appears to be an important determinant of synovial MSC properties. The mechanistic basis for this association is explored in Section 4.5.

### 4.5. Molecular Mechanisms Underlying Source-Dependent Heterogeneity in MSC Properties

A consistent theme across all three comparison domains is that the properties of synovial MSCs appear to reflect their respective microenvironments. Evidence supporting this includes the convergence of immunophenotypes after expansion [28], the reduction in proliferative differences upon clonal selection compared to mixed culture [24] and the apparent influence of isolation method on MSC properties [26]. The underlying molecular mechanisms warrant further discussion, with proximity to vasculature and a peri-vascular niche appearing to be recurrent determinants of MSC properties.

MSCs have been proposed to originate from pericytes, with established literature demonstrating that pericytes express MSC-adjacent markers and have trilineage differentiation potential comparable to MSCs [33]. Amongst the studies included in this review, MSCs from the perivascular fraction of SM had the highest proliferative, chondrogenic and osteogenic potential [28], whilst CFE across harvest sites correlated directly with local vascular density [29,30]. A candidate molecular mechanism is paracrine trophic signalling from endothelial cells and pericytes to adjacent MSCs. Amemiya et al. [21] demonstrated that CD140b (the receptor for PDGFβ, an endothelium-derived mitogen) was more strongly expressed in SM-MSCs compared to those from SF and that its expression correlated with the proliferation rates of both MSC sources. As discussed, SF-MSCs are believed to originate from the SM and are mobilised in response to injury [17,18]. Upon mobilisation, the loss of this paracrine support is a coherent explanation for the reduced proliferative capacity of SF-MSCs compared to those from SM, despite their shared origin. Consistent with a dynamic relationship between endothelial cells and MSCs, human aortic endothelial cells have been shown to direct the osteogenic and chondrogenic differentiation of co-cultured bone marrow MSCs through AKT and endothelin-1 signalling [34]. Such a mechanism may also be operating in the synovial-perivascular niche microenvironment.

Paracrine signalling in the perivascular niche is unlikely to be confined to PDGF signalling. Other candidate molecular mediators of the differences observed include the CXCL12-CXCR4 axis. In bone marrow perivascular niches, high levels of CXCL12 expression have been identified on perivascular cells, and disruption of this signalling pathway via knockout of CXCR4 significantly reduces the proliferation potential of haematopoietic stem cells [35]. Similar mechanisms operating in the synovial joint could again explain the heterogeneity of MSC properties observed, with mobilisation of MSCs into synovial fluid reducing their exposure to these paracrine factors.

Notch signalling is another potential molecular mediator that warrants discussion, again potentially contributing to the observed spatial heterogeneity of SM- MSC properties [28,30]. In synovial sections from patients with rheumatoid arthritis, endothelium-derived Notch signalling has been shown to influence the differentiation of CD90-expressing fibroblasts [36]. Such signalling could also be operating to influence the properties of MSCs in the synovial microenvironment.

Differences in properties between FS- and AS- MSCs may also be explained by source-specific transcriptional priming. The greater adipogenic differentiation potential of AS-MSCs, along with greater expression of adipsin, PPAR-γ, and LPL observed by Nakashima et al. [27] is consistent with these MSCs being transcriptionally primed by their lipid-rich adipose surrounding stroma, compared to the collagen-rich environment of FS-MSCs. Differences in the composition of the extracellular matrix and local growth-factor milieu may bias the differentiation potential of MSCs from these different sources.

Importantly, however, such environmental priming appears not to be fixed, but plastic, with differences between FS and AS-MSCs attenuating with isolation methods and clonal selection [24,26]. It is well-established in the broader MSC literature that prolonged culture is associated with telomere shortening, replicative senescence, and the progressive loss of canonical markers like CD146 and CD140b [32,37].

Taken together, these data suggest that the heterogeneity observed in synovial-MSC properties is likely a reflection of the continuum of microenvironments these cells can exist in, with proximity to vasculature and perivascular trophic signalling appearing to have a large influence. Further elucidating these molecular mechanisms will require experimental approaches which can capture the dynamic spatial nature of paracrine trophic signalling with single-cell resolution, which are subsequently detailed in Section 4.6.

### 4.6. Directions for Future Research

There are several priorities for future research on synovial MSCs arising from this review. First, the limited and heterogeneous in vitro evidence base should be strengthened through adequately powered, donor-matched studies applying quantitative and standardised assays. Beyond refining existing approaches, there are a number of emerging molecular techniques that offer the opportunity to further elucidate the molecular basis of differences in MSC properties, which requires further discussion.

Single-cell approaches are particularly important for this field of study. Single-cell RNA sequencing (scRNA-seq) and combined surface proteomic and transcriptomic methods, such as CITE-seq [38], could help elucidate whether different synovial sources comprise truly distinct MSC populations, or instead comprise a shared continuum of cells. Such methods could also help more precisely assess the aforementioned clonal drift seen in prolonged culture [32,37]. These methods have been employed to evaluate the heterogeneity of cellular populations within synovial joints of mice, where the use of scRNA-seq has facilitated the creation of a transcriptomic atlas of the synovium and the identification of distinct progenitor populations in a sublining perivascular niche [39].

A key finding of Amemiya et al. [21] was the distinct phenotypic identities of SF- and SM-MSCs beyond the standard ISCT markers. ScRNA-seq has been employed to evaluate the surface expression of MSCs from bone marrow and Wharton’s Jelly, identifying subsets of MSCs with distinct phenotypes and subsequently functional properties, including proliferation and stemness [40]. Applying these approaches to donor-matched synovial MSC sources could enable further phenotypic and functional stratification of these cells, beyond the current limited ISCT marker set used to identify these populations.

These single-cell approaches could be complemented by spatial approaches, which preserve the anatomical context of the synovial joint. The perivascular niche hypothesis could be tested via spatial transcriptomics of intact synovial sections, mapping ligand and receptor distributions. Such approaches have precedence in the literature and have been used to identify the influence of endothelium-derived Notch signalling on fibroblast differentiation in joints affected by rheumatoid arthritis [36]. Further integrating these data with additional multi-omic approaches, such as proteomics and epigenomics, could help to differentiate between stable, heritable MSC properties from those that are transient and arise from the local microenvironment.

Epigenetic approaches are particularly suited for this application. DNA-methylation status has been shown to vary not just between MSCs and non-MSC sources like fibroblasts, but also between MSCs from adipose tissue and bone marrow [41]. Chromatin accessibility is another important layer of epigenetic regulation, and techniques such as Assay for Transposase-Accessible Chromatin using sequencing (ATAC-seq) have demonstrated that MSCs from different sources, like bone marrow and adipose tissue, have distinct epigenetic profiles [42].

Ultimately, these multi-omic methods should be applied to donor-matched synovial sources to help further elucidate whether these different MSC sources are truly distinct populations or whether observed differences in properties arise from heterogeneity in the local microenvironments.

### 4.7. Limitations of Included Studies

Whilst the results of the studies included in this review are encouraging, there is also methodological heterogeneity to be wary of, including the age and disease status of donors, culture conditions and outcome measures assessed.

One limitation in particular is the reliance on qualitative or semi-quantitative assessments of differentiation potential. Most studies only performed semi-quantitative analyses of samples post-differentiation as opposed to performing rigorous quantification of differentiation products, like GAG or collagen content. This may have masked underlying differences in differentiation potential between sources. Future studies should employ standardised quantitative differentiation assays to ensure meaningful cross-study comparison.

Another key limitation in studies excluded during full-text screening was the lack of donor-matching between synovial sources, with several studies otherwise meeting the inclusion criteria but being excluded for this reason. This is a necessary exclusion criterion to control for confounding by inter-donor variability, with evidence of the potential influence of age on the proliferation characteristics of FS- and AS-MSCs being a clear demonstration of its importance [24].

Another limitation is the lack of in vivo evidence. Only one study performed any kind of in vivo assessment of the properties of synovial MSCs [21]. Especially considering the aforementioned potential influence of culture conditions on MSC properties, it is vital to conduct more in vivo studies to see whether findings in vitro will be of benefit to patients.

A further limitation of our own review methodology is the restriction to full-text articles published in the English language, which introduces language bias. This may have resulted in eligible donor-matched comparisons of synovial-MSC sources being missed, and hence the body of literature synthesised here may not be representative of the global body of evidence. This is particularly relevant to the field of synovial-MSC research, where relevant research is published in Japanese-language journals. Future reviews in this area would thus benefit from including non-English language studies, with translation where necessary, to reduce the selection bias.

### 4.8. Clinical Implications

Transplanted synovial MSCs have also been shown to be efficacious in repairing cartilage defects [43], and the findings of this review may have implications for the source of MSCs for these applications.

MSCs used in such applications would ideally have high proliferative capacity, robust trilineage differentiation potential and be practically accessible, with minimal donor-site morbidity [44]. The superior or at least non-inferior proliferative characteristics of SM-MSCs may make these a more suitable source than those from SF in this regard, although differentiation potential was seen to be broadly equivalent between these two sources. However, donor-site morbidity would be markedly less for harvesting SF-MSCs, and proliferation differences were not unequivocally shown in all included studies.

Within the SM, the superior proliferative and chondrogenic potential of MSCs from the perivascular fraction may make this the most appropriate source. The heterogeneity regarding differences in FS- and AS-MSC properties warrants caution when making conclusions regarding their relative suitability for regenerative therapies.

Ultimately, the current evidence base of pre-clinical, primarily in vitro studies, is not sufficient to make conclusions regarding the ideal source of synovial MSCs for regenerative applications. More in vivo work, akin to the osteochondral defect repair conducted by Amemiya et al. [21], would help to resolve this. However, these preliminary findings do suggest that the different synovial-MSC sources may be of varying therapeutic utility for regenerative purposes.

## 5. Conclusions

It is well established in the literature that synovium-derived MSCs have superior proliferative and chondrogenic properties compared to MSCs from other sources, like adipose and bone marrow [15].

This review further adds to this field by demonstrating that synovial MSCs are heterogeneous in their properties. Synovial MSCs can be isolated from SF and SM, FS and AS, and different anatomical regions within the SM. These MSCs are heterogeneous in their proliferative and differentiation potentials and their immunophenotypes. Heterogeneity in the properties of synovial MSCs appears to be driven by differences in their respective local microenvironments, rather than there being separate populations of MSCs with intrinsically different properties. Proximity to vasculature, in particular, is one such environmental factor which appears to influence MSC properties. These findings may have implications for the choice of MSCs for cell-based regenerative therapies, although more work is required to validate these potential differences and elucidate their mechanistic basis.

## Figures and Tables

**Table 1:** General characteristics of the ten included studies.

Study	Joint	Donors (N; Sex; Age)	Disease Status of Donors	MSC Sources Compared	Outcomes Reported
Amemiya et al. 2020 [21]	Knee	14 (13F/1M); mean age 73 y (58–83)	End-stage knee OA (KL III *n* = 1; KL IV *n* = 13)	SF (arthrocentesis) vs. SM (suprapatellar pouch)	Proliferation, CFE, cell cycle, 242-marker Lyoplate panel for immunophenotype, in vitro trilineage differentiation potential (qualitative) and in vivo rat osteochondral-defect repair
Ferro et al. 2019 [22]	Knee	19 (10F/9M); age 20–78 y	Mixed (traumatic injury in younger donors; OA in older donors)	SM (biopsied during routine arthroscopy) vs. SF	Growth kinetics, CPD, CFE, trilineage differentiation potential (qualitative; SF not tested), immunophenotype
Lee et al. 2012 [23]	Knee	28 (24F/4M); mean age 56 y (43–76)	Primary knee OA (KL I *n* = 3; II *n* = 7; III *n* = 10; IV *n* = 8)	SF (aspiration) vs. SM (suprapatellar pouch during arthroscopic meniscectomy or TKA)	MSC proportion (CD34^−^/CD44^+^/CD90^+^), including proportion stratified by KL score, individual ISCT-relevant markers, in vitro trilineage differentiation potential (qualitative)
Mochizuki et al. 2006 [24]	Knee	8 (4 young, 4 elderly); young mean age 17.2 ± 0.7 y; elderly mean age 70.5 ± 9.2 y; sex not reported	Young = ACL injury; elderly = knee OA	FS (lateral joint capsule) vs. AS (inner IPFP) vs. subcutaneous fat (SCF)	Nucleated-cell yield, surface antigens, proliferation (mixed and single-clone), CFE, in vitro trilineage differentiation potential (quantitative)
Nakashima et al. 2022 [27]	Ankle	14 CLAI without OLTs (5F/9M; mean age 33.3 ± 19.4 y) + 6 CLAI with OLTs (mean age 38.4 ± 14.0 y)	Chronic lateral ankle instability (CLAI), refractory to conservative management	ATFL-surrounding FS vs. anterior fat pad (AFP, adipose synovium); SF	Yield, CFE, expandability, viability, in vitro trilineage differentiation potential (colony rates + qPCR), flow cytometry for immunophenotype
Katagiri et al. 2017 [25]	Knee	34 (28 single-arm + 6 paired FS vs. AS; paired subset 5F/1M, mean age 75 y)	Knee OA, TKA	FS (suprapatellar pouch) vs. AS (inner IPFP)	Colony number (paired FS vs. AS, *n* = 6), cell yield, immunophenotype (FS only), in vitro trilineage differentiation potential (FS only, qualitative)
Lee et al. 2011 [26]	Knee	40 (35F/5M); mean age 67 y (58–81)	Knee OA, TKA	FS (suprapatellar pouch + lateral joint capsule) vs. AS (inner IPFP)	Cell yield, MSC proportion (CD34^−^/CD44^+^/CD90^+^), immunophenotype, in vitro trilineage differentiation potential (qualitative)
Nagase et al. 2008 [30]	Knee	10 TKA donors; age 63–91 y; sex not reported (*n* = 3–5 per experiment)	Medial-compartment knee OA (femorotibial angle 185–195°)	Four intra-articular harvest sites: medial outer capsule, suprapatellar pouch, IPFP, medial inner condyle	Vessel density (α-SMA^+^, CD31^+^), CFE, surface markers during P0, in vitro trilineage differentiation potential (chondrogenic-pellet weight and GAG, adipogenic/calcification colony rates)
Mizuno et al. 2018 [28]	Knee	10 TKA donors (IHC *n* = 6; FACS ratio *n* = 8; proliferation/colony/differentiation *n* = 4)	Knee OA	Surface (CD55^+^/CD271^−^), stromal (CD55^−^/CD271^−^), and perivascular (CD55^−^/CD271^+^) sorted fractions of synovial membrane	IHC of 19 markers across regions, sort-fraction ratios, proliferation, CFE, surface markers (P3), trilineage differentiation potential (quantitative + qPCR)
Murata et al. 2018 [29]	Hip	18 FAIS donors (11F/7M); mean age 41.8 ± 16.2 y (19–64)	FAIS; OA, inflammatory/traumatic/infectious/necrotic and prior-surgery cases excluded	Paralabral synovium (5 mm adjacent to labral tear) vs. cotyloid-fossa synovium	Yield, CFE, expandability, viability, assessment of ISCT-relevant immunophenotypic markers, in vitro trilineage differentiation potential (quantitative)

Abbreviations: ACL = anterior cruciate ligament; AFP = anterior fat pad; α-SMA = α-smooth muscle actin; ATFL = anterior talofibular ligament; CFE = colony-forming efficiency; CLAI = chronic lateral ankle instability; CPD = cumulative population doublings; F = female; FACS = fluorescence-activated cell sorting; FAIS = femoroacetabular impingement syndrome; GAG = glycosaminoglycan; ICRS = International Cartilage Repair Score; IHC = immunohistochemistry; IPFP = infrapatellar fat pad; ISCT = International Society for Cell and Gene Therapy; KL = Kellgren–Lawrence; M = male; OA = osteoarthritis; OLT = osteochondral lesion of the talus; P = passage; qPCR = quantitative polymerase chain reaction; TKA = total knee arthroplasty.

**Table 2:** Synovial fluid (SF) vs. synovial membrane (SM) MSCs—cellular yield, clonogenicity and proliferation.

Study	Cellular Yield, Clonogenicity and Proliferation
Amemiya et al. 2020 [21]	SM is consistently superior. P0 primary-cell yield: SM 2.9 × 10^6^ vs. SF 4.7 × 10^5^ per sample (~6-fold lower; *p* < 0.001). Colonies at P2–P4: SM ~35 vs. SF ~20 per 60 cm^2^ dish (*p* < 0.05). Population doubling time P1–P2: SM 2.5 d vs. SF 4.5 d (*p* < 0.05), maintained to P9–P10 (SM 3.5 d vs. SF 5.0 d). Proportion of cells in S-phase of cell cycle: SM ~20% vs. SF ~10% (*p* < 0.05). CD140a and CD140b expression inversely correlated with doubling time (*r* = −0.77, *p* = 0.010; *r* = −0.82, *p* = 0.004).
Lee et al. 2012 [23]	SM-MSC proportion > SF-MSC at initial harvest, but the gap narrows with expansion. MSC proportion (CD34^−^/CD44^+^/CD90^+^): P0 SF 1.11 ± 1.22% vs. SM 5.00 ± 2.29% (*p* = 0.019); P1 SF 49.65 ± 17.72% vs. SM 57.58 ± 15.97% (*p* = 0.759); P2 SF 59.21 ± 19.47% vs. SM 70.48 ± 15.16% (*p* = 0.936). Difference in proliferation rate between SM- and SF-MSCs across P0→P2: NS (F_2,26_ = 1.78; *p* = 0.188). SF-MSC proportion at P0 declined with disease severity (KL I 1.82% → KL IV 0.48%; *p* = 0.033; *r* = −0.565, *p* = 0.002); KL grade independent predictor on multivariable regression (β = −0.356, *p* = 0.039). SM-MSC proportion is independent of KL at every passage.
Ferro et al. 2019 [22]	SM-MSCs appear to have superior proliferative characteristics compared to those from SF. CPD at P8: SM 13 ± 0.73 > SF 11 ± 0.97 (significance of this difference not tested). Population-doubling time P1–P3: SM 160 ± 33 h < SF 180 ± 52 h (significance of this difference not tested).

Abbreviations: PDGFR = platelet-derived growth-factor receptor. NS denotes where a between-source comparison was formally tested and was not found to be statistically significant—where no statistical test was performed between sources, this is explicitly stated.

**Table 3:** Synovial fluid (SF) vs. synovial membrane (SM) MSCs—immunophenotype.

Study	Immunophenotype
Amemiya et al. 2020 [21]	ISCT-panel markers broadly comparable: CD73 and CD90: NS. CD105: SM 99.80 ± 0.09% vs. SF 99.00 ± 0.72% (*p* = 0.043; small but significant). Residual CD34 was higher in SM (23.70 ± 21.50% vs. SF 2.65 ± 3.38%; *p* = 0.043). Comprehensive 242-marker Lyoplate panel identified a cluster of markers significantly higher in SM (all SM vs. SF; *p* < 0.05): CD106/VCAM-1: 9.59 ± 6.39 vs. 3.42 ± 1.78%; CD107a: 21.10 ± 11.50 vs. 5.44 ± 3.54%; CD121a: 10.60 ± 8.06 vs. 1.62 ± 0.86%; CD140b/PDGFRβ: 99.10 ± 1.07 vs. 77.90 ± 24.70%; CD141: 63.70 ± 24.00 vs. 28.80 ± 32.30%; CD143: 75.40 ± 9.23 vs. 30.00 ± 28.90%.
Lee et al. 2012 [23]	Individual-marker flow at P1: CD34^−^: SF 81.35% vs. SM 97.48%; CD44: SF 97.71% vs. SM 95.94%; CD90: SF 87.35% vs. SM 75.88%. Formal between-source statistical testing not reported per individual marker; overall ISCT-relevant profile broadly comparable, with higher residual CD34 positivity in SF (i.e., lower CD34 negativity)—directionally opposite to Amemiya et al. 2020 [21], although no formal statistical significance of this difference was reported.
Ferro et al. 2019 [22]	The ISCT profile is broadly conserved across sources. CD90: SM 84–97; SF: 83–86%. CD105: SM max 90.2%; SF range 77–87% (SF CD105 lower and more variable, but difference vs. SM not formally tested). CD14, CD19, CD34, CD45, CD80 and HLA-DR all <2% in all sources.

Abbreviations: HLA-DR = human leukocyte antigen-DR; PDGFRβ = platelet-derived growth-factor receptor β; VCAM-1 = vascular cell-adhesion molecule 1. NS denotes where a between-source comparison was formally tested and was not found to be statistically significant—where no statistical test was performed between sources, this is explicitly stated.

**Table 4:** Synovial fluid (SF) vs. synovial membrane (SM) MSCs—differentiation potential.

Study	Differentiation Potential
Amemiya et al. 2020 [21]	Trilineage differentiation potential confirmed by qualitative staining in both sources, with no significant differences between SM and SF on clonal analysis. In vivo assessment (rat osteochondral defect, 1.4 mm; outcome is ICRS at 8 weeks): SM 8, SF 9, PBS ~1—both MSC sources significantly better than PBS (*p* < 0.05); no difference between SM and SF.
Lee et al. 2012 [23]	Both SF and SM cultures differentiated successfully into adipogenic, osteogenic and chondrogenic lineages following induction. No numerical data or quantitative between-source comparison reported.

Abbreviations: PBS = phosphate-buffered saline.

**Table 5:** Fibrous synovium (FS) vs. adipose synovium (AS) MSCs—cellular yield, clonogenicity and proliferation.

Study	Cellular Yield, Clonogenicity and Proliferation
Mochizuki et al. 2006 [24]	Nucleated-cell yield per mg is broadly equivalent between FS and AS. Mixed-population proliferation (fold increase over 14 d): FS young ~100× vs. AS young ~60× (*p* < 0.05, two-factor ANOVA)—difference absent in elderly donors and lost when single-clone populations were compared. CFE (mixed-population): FS young ~80% vs. AS young ~65% (NS between FS and AS; both *p* < 0.05 vs. SCF ~20%). Clonal CFE: FS ~90%, AS ~75%, SCF ~20% (*p* < 0.005 vs. SCF).
Nakashima et al. 2022 [27]	Broadly equivalent proliferation between ATFL-surrounding fibrous synovium and AFP. No. of nucleated cells per mg: ATFL 1.9 ± 2.3 × 10^4^ vs. AFP 1.4 ± 1.6 × 10^4^ (*p* = 0.272). No. of colonies: ATFL 60.1 ± 56.4 vs. AFP 99.8 ± 123.8 (*p* = 0.722). Cells per colony: ATFL 4.0 ± 3.5 vs. AFP 4.5 ± 7.2 × 10^4^ (*p* = 0.929). P0 yield: ATFL 1.9 ± 2.9 vs. AFP 2.9 ± 3.7 × 10^4^ (*p* = 0.099). Expandability lost at P8 for both sources; P4 viability 100% ATFL vs. 94% AFP; P7 49% vs. 46%. Comparator CLAI-with-OLTs donors yielded significantly more colonies for both sources than those without OLTs (*p* = 0.009 ATFL, *p* = 0.037 AFP). Similar cellular yield was maintained across all passages.
Katagiri et al. 2017 [25]	FS > AS for colony release. In the suspended-synovium culture model, FS yielded more colonies per 1 g of tissue than AS in every individual donor.
Lee et al. 2011 [26]	Nucleated cells per g pre-expansion: FS 8.80 ± 7.53 vs. AS 7.11 ± 7.14 × 10^5^ (*p* = 0.58). After 10 d of expansion (×10^5^/g): Coll-FS 4.88 ± 4.35; Coll-AS 3.89 ± 5.93; Expl-FS 3.12 ± 3.61; Expl-AS 2.58 ± 1.88 (all NS). MSC proportion at isolation: FS 4.54 ± 4.05% vs. AS 2.58 ± 2.36% (*p* < 0.05). MSC proportion after expansion (CD34^−^/CD44^+^/CD90^+^): Coll-FS 52.86 ± 19.80%; Coll-AS 63.59 ± 13.40%; Expl-FS 68.21 ± 20.55%; Expl-AS 72.26 ± 15.71% (enzymatic vs. explant *p* < 0.05 for FS). Fold increase in MSC proportion over 10 d: Coll-AS 24.1 ± 5.2 vs. Coll-FS 11.1 ± 3.5—AS expanded more when enzymatic digestion was used.

Abbreviations: ANOVA = analysis of variance; Coll = collagenase-digested; Expl = direct-explant; SCF = subcutaneous fat. NS denotes where a between-source comparison was formally tested and was not found to be statistically significant—where no statistical test was performed between sources, this is explicitly stated.

**Table 6:** Fibrous synovium (FS) vs. adipose synovium (AS) MSCs—immunophenotype.

Study	Immunophenotype
Mochizuki et al. 2006 [24]	The ISCT profile was broadly conserved between FS and AS. CD45/CD31/CD117/CD34 all <2% in FS and AS. CD44 70–90%, CD90 40–70%, CD105 60–80%, CD147 > 90%—comparable between FS and AS across donors. FS and AS distinguished from SCF by STRO-1 (>6% vs. 1–2%; *p* < 0.05), CD106 (5% vs. 1%; *p* < 0.05) and lower CD10 (10% vs. ~40%; *p* < 0.05).
Nakashima et al. 2022 [27]	ISCT profile conserved between sources; both had unusually low CD105 expression compared to knee-derived MSCs (ATFL 8.5% vs. AFP 6.3%; *p* = 0.593). CD44: ATFL 49.4% vs. AFP 41.0% (*p* = 0.068); CD90: 89.8% vs. 89.2% (*p* = 0.715); CD147: 10.2% vs. 12.5% (*p* = 0.465). CD31, CD34, CD45, CD117, VEGFR-2 and NGFR all <2% in both sources.
Katagiri et al. 2017 [25]	FS only sampled, AS not profiled for immunophenotype.
Lee et al. 2011 [26]	Individual ISCT-relevant markers at P1: CD34^−^ ≥ 94% all four groups; CD44^+^ > 97% all four groups (Coll-AS 99.23%, Coll-FS 97.95%, Expl-AS 98.80%, Expl-FS 99.86%); CD90^+^ > 99% all four groups; CD105^+^ > 97% all four groups (MFI for Expl-FS significantly higher than the other three groups, *p* < 0.05). Overall, FS and AS immunophenotypes were highly conserved.

Abbreviations: MFI = median fluorescence intensity; NGFR = nerve-growth-factor receptor; STRO-1 = stromal precursor antigen-1; VEGFR = vascular endothelial growth-factor receptor. NS denotes where a between-source comparison was formally tested and was not found to be statistically significant—where no statistical test was performed between sources, this is explicitly stated.

**Table 7:** Fibrous synovium (FS) vs. adipose synovium (AS) MSCs—differentiation potential.

Study	Differentiation Potential
Mochizuki et al. 2006 [24]	Chondrogenic pellet wet weight (mg): FS young ~1.5, AS young ~1.2, SCF young ~0.3 (FS and AS both *p* < 0.05 vs. SCF; no significant difference between FS and AS). C4S content (μg/pellet): FS, ~2.0 AS ~1.5 (NS). Alizarin red-positive colony rate: FS ~80%, AS ~75% (*p* < 0.05 vs. SCF ~40%). Oil Red O-positive colony rate: FS ~75% vs. AS ~70% (NS). Chondrogenic gene expression (*COL2A1*, *SOX9*, *SOX6*, *aggrecan*) is higher in FS and AS vs. SCF; no FS-vs-AS difference.
Nakashima et al. 2022 [27]	AS (AFP) shows selectively greater adipogenic potential. Oil Red O-positive colony rate: ATFL ~30% vs. AFP ~65% (*p* = 0.028). Adipsin mRNA was 2.5-fold higher in AFP (*p* < 0.05); *PPAR-γ* was 4-fold, and *LPL* was 5-fold higher in AFP, but did not reach significance. Osteogenic (ALP^+^/vK^+^) colony rate: ATFL ~55% vs. AFP ~60% (*p* = 0.866); osteogenic mRNA (*COL1A1*, *ALPP*, *RUNX2*, *BGLAP*) all NS. Chondrogenic qPCR at P3 pellet *COL2A1*, *SOX9*, *COL10A1*: all NS (*p* = 0.188–0.438).
Katagiri et al. 2017 [25]	Paired FS vs. AS differentiation comparison was not performed.
Lee et al. 2011 [26]	Trilineage differentiation was confirmed qualitatively in all four groups (Coll-FS, Coll-AS, Expl-FS, Expl-AS) after induction in adipogenic, osteogenic and chondrogenic media. No quantitative between-source comparison performed.

Abbreviations: ALP = alkaline phosphatase; *ALPP* = alkaline phosphatase (placental); *BGLAP* = bone gamma-carboxyglutamate protein (osteocalcin); C4S = chondroitin-4-sulfate; *COL* = collagen; *LPL* = lipoprotein lipase; mRNA = messenger RNA; *PPAR-γ* = peroxisome proliferator-activated receptor γ; *RUNX2* = runt-related transcription factor 2; *SOX* = SRY-box transcription factor; vK = von Kossa.

**Table 8:** Intra-articular regional variation in synovial MSCs—clonogenicity, proliferation and vascular association.

Study	Regions Compared	Clonogenicity, Proliferation & Vascular Association
Nagase et al. 2008 [30]	Four intra-articular harvest sites within the knee synovium: medial outer capsule, suprapatellar pouch, infrapatellar fat pad, and medial inner condyle.	CFE varied ~4-fold by harvest site. The medial outer capsule had the highest (~40–50 colonies per 10^3^ nucleated cells) and the medial inner had the lowest (10–20 per 10^3^; *p* < 0.05 Friedman). α-SMA+ vessel density was highest at the medial outer capsule (80–100/mm^2^) and lowest at the medial inner (15–25/mm^2^; Kruskal–Wallis *p* < 0.05). CD31+ cell density: medial outer ~1000–1500/mm^2^ vs. other sites ~150–400/mm^2^ (*p* = 0.0004). Both α-SMA+ and CD31+ correlated positively with CFU number (Spearman *p* < 0.05). Cells per colony at days 7/14/21 did not differ between sites.
Mizuno et al. 2018 [28]	FACS-sorted sub-fractions of knee synovial membrane: surface (CD55+/CD271−), stromal (CD55−/CD271−) and perivascular (CD55−/CD271+).	Perivascular fraction proliferated most: ~150–200-fold over 10 d vs. ~50–70-fold for surface and stromal fractions (*p* < 0.01). Colony number per well: 10–15 across all five fractions (NS). Fraction abundance of non-haematopoietic cells: surface ~5%, stromal ~70%, perivascular ~15% (stromal > surface and perivascular, *p* < 0.01). IHC (perivascular regions) showed the highest staining for proliferation markers Ki67 and PCNA, and vascular markers CD31, CD146, collagen IV and laminin.
Murata et al. 2018 [29]	Two intra-articular regions of hip synovium: paralabral synovium (5 mm adjacent to labral tear) vs. cotyloid-fossa synovium.	Cotyloid fossa strongly outperformed paralabrum. CFU per 10^4^ nucleated cells: paralabral ~9.0 ± 15.7 vs. cotyloid ~31.6 ± 23.7 (3.5-fold; *p* < 0.01). Yield at P0: paralabral ~1.1 ± 2.6 vs. cotyloid ~12.1 ± 27.3 (11-fold; *p* < 0.01). Expandability: lost at P10 for paralabral vs. P14 for cotyloid. Sample weight and cells per mg were not significantly different between regions.

NS denotes where a between-source comparison was formally tested and was not found to be statistically significant—where no statistical test was performed between sources, this is explicitly stated.

**Table 9:** Intra-articular regional variation in synovial MSCs—immunophenotype.

Study	Regions Compared	Immunophenotype
Nagase et al. 2008 [30]	Four intra-articular harvest sites within the knee synovium: medial outer capsule, suprapatellar pouch, infrapatellar fat pad, and medial inner condyle.	During P0 expansion of suprapatellar-pouch-derived MSCs, several markers declined significantly over 7–21 d: CD105 from 95–100% to 40–60% (*p* < 0.05); CD166 from 50–80% to 5–15% (*p* < 0.05); CD90 from 40–60% to 10–20% (*p* < 0.05); CD55 from 20–30% to 5–10% (*p* < 0.05). STRO-1 remained ~40–60% across timepoints. CD54 and CD106 remained stable (NS). CD68 undetectable.
Mizuno et al. 2018 [28]	FACS-sorted sub-fractions of knee synovial membrane: surface (CD55+/CD271−), stromal (CD55−/CD271−) and perivascular (CD55−/CD271+).	Fresh-sort immunophenotype distinguished the three fractions, but these differences had converged by P3. At P3, CD44+, CD73+ and CD90+ were all >90% across fractions; CD271+ ≤ 10% in all fractions (i.e., the perivascular marker was lost with expansion); CD31, CD45, and CD146 were all <3%. Surface fraction retained higher CD55, PRG4, HAS-1 and HAS-2 on IHC; stromal fraction high CD73; perivascular fraction high CD105, CD271, CD140a, CD140b, CD29, CD49f.
Murata et al. 2018 [29]	Two intra-articular regions of hip synovium: paralabral synovium (5 mm adjacent to labral tear) vs. cotyloid-fossa synovium.	Flow panel at P3 (paralabral vs. cotyloid): CD44 87.2% vs. 66.4%; CD90 93.5% vs. 89.4%; CD105 43.4% vs. 31.4%; CD147 73.4% vs. 65.4%. CD45, CD31, CD117, CD34, CD166, VEGFR-2, NGFR all negative. Formal per-marker statistical testing not reported; overall ISCT-relevant profile comparable between regions.

NS denotes where a between-source comparison was formally tested and was not found to be statistically significant—where no statistical test was performed between sources, this is explicitly stated.

**Table 10:** Intra-articular regional variation in synovial MSCs—differentiation potential.

Study	Regions Compared	Differentiation Potential
Nagase et al. 2008 [30]	Four intra-articular harvest sites within the knee synovium: medial outer capsule, suprapatellar pouch, infrapatellar fat pad, and medial inner condyle.	Chondrogenic-pellet wet weight was similar across the four harvest sites (NS between sites). Adipogenic CFE ~60% and calcification CFE ~30% regardless of harvest site or preculture period.
Mizuno et al. 2018 [28]	FACS-sorted sub-fractions of knee synovial membrane: surface (CD55+/CD271−), stromal (CD55−/CD271−) and perivascular (CD55−/CD271+).	Perivascular > surface/stromal for chondrogenic and osteogenic differentiation. Chondrogenic pellet diameter ~2.0–2.5 mm perivascular vs. ~0.8–1.2 others (*p* < 0.01); SOX9 mRNA ~8–10× higher (*p* < 0.01 vs. bulk/stroma); ACAN ~4–5× (*p* < 0.01 vs. stroma); COL10A1 ~70–100× higher (*p* < 0.01). Calcification: alizarin red absorbance 3–4× higher in perivascular (*p* < 0.05 vs. all); ALP and RUNX2 mRNA NS. Adipogenesis NS between fractions (triglyceride, PPARG, LPL NS); GTF3A and CEBPA were lower in surface (*p* < 0.05 vs. other four fractions).
Murata et al. 2018 [29]	Two intra-articular regions of hip synovium: paralabral synovium (5 mm adjacent to labral tear) vs. cotyloid-fossa synovium.	Cotyloid > paralabrum across all three lineages. Oil Red O-positive colony rate: paralabral 45.4 ± 15.1% vs. cotyloid 64.5 ± 9.1% (*p* < 0.05). Adipogenic mRNA (fold vs. paralabral = 1): Adipsin 11.0 ± 1.5 (*p* < 0.01); LPL 3.6 ± 0.7 (*p* < 0.01); PPARG 2.9 ± 0.6 (*p* < 0.01). Osteogenic von Kossa+/ALP+ colony rate: paralabral 39.7 ± 11.2% vs. cotyloid 59.8 ± 12.5% (*p* < 0.05). BGLAP mRNA 5.2-fold higher in cotyloid (*p* < 0.05); COL1A1, ALPP, RUNX2 NS. Chondrogenic qPCR: COL2A1 19.9-fold higher in cotyloid (*p* < 0.01); SOX9 1.4-fold higher (*p* < 0.01); COL10A1 NS.

NS denotes where a between-source comparison was formally tested and was not found to be statistically significant—where no statistical test was performed between sources, this is explicitly stated. Abbreviations (Table 8, Table 9 and Table 10): ACAN = aggrecan; CEBPA = CCAAT/enhancer-binding protein alpha; CFU = colony-forming unit; GTF3A = general transcription factor IIIA; HAS = hyaluronan synthase; PCNA = proliferating-cell nuclear antigen; PRG4 = proteoglycan 4.

**Table 11:** OHAT risk-of-bias assessment for the ten included human studies.

Risk of Bias Domain	1	2	3	4	5	6	7	8	9	10
Randomisation of administered dose or exposure level	N/A	N/A	N/A	N/A	N/A	N/A	N/A	N/A	N/A	N/A
Allocation concealment	N/A	N/A	N/A	N/A	N/A	N/A	N/A	N/A	N/A	N/A
Appropriate participant selection for comparison	++	+	++	++	++	+	++	+	+	++
Accounting for important confounding/modifying variables	+	−	+	+	+	−	+	+	−	+
Identical experimental conditions across study groups	++	++	++	++	++	++	++	++	++	++
Blinding of research personnel	−	−	−	−	−	−	−	−	−	−
Incomplete outcome data	+	−	++	++	+	+	+	+	+	+
Confidence in exposure characterisation	++	+	++	++	++	++	++	++	++	++
Confidence in outcome assessment (incl. assessor blinding)	+	−	−	−	−	+	−	−	−	−
Complete reporting of measured outcomes	++	+	++	++	++	+	++	+	+	++
Other potential threats to internal validity	++	−	+	++	++	+	+	++	−	+

Study ID: 1 = Amemiya et al. (2020) [21]; 2 = Ferro et al. (2019) [22]; 3 = Lee et al. (2012) [23]; 4 = Mochizuki et al. (2006) [24]; 5 = Nakashima et al. (2022) [27]; 6 = Katagiri et al. (2017) [25]; 7 = Lee et al. (2011) [26]; 8 = Nagase et al. (2008) [30]; 9 = Mizuno et al. (2018) [28]; 10 = Murata et al. (2018) [29]. Legend: ++ = definitely low risk; + = probably low risk; − = probably high risk; N/A = not applicable.

## Data Availability

No new data were created or analysed in this study. Data sharing is not applicable to this article.

## Abbreviations

The following abbreviations are used in this manuscript:

ACAN	Aggrecan
ACL	Anterior cruciate ligament
AFP	Anterior fat pad
AKT	AKT serine/threonine kinase (protein kinase B)
ALP	Alkaline phosphatase
ALPP	Alkaline phosphatase (placental)
ANOVA	Analysis of variance
AS	Adipose synovium
ATFL	Anterior talofibular ligament
BGLAP	Bone gamma-carboxyglutamate protein (osteocalcin)
C4S	Chondroitin-4-sulfate
CD	Cluster of differentiation
CEBPA	CCAAT/enhancer-binding protein alpha
CFE	Colony-forming efficiency
CFU	Colony-forming unit
CITE-seq	Cellular Indexing of Transcriptomes and Epitopes by sequencing
CLAI	Chronic lateral ankle instability
Coll	Collagenase-digested
COL	Collagen (e.g., COL1A1, COL2A1, COL10A1)
CPD	Cumulative population doublings
CXCL12	C-X-C motif chemokine ligand 12 (stromal cell-derived factor 1)
CXCR4	C-X-C motif chemokine receptor 4
Expl	Direct-explant
F	Female
FACS	Fluorescence-activated cell sorting
FAIS	Femoroacetabular impingement syndrome
FS	Fibrous synovium
GAG	Glycosaminoglycan
GTF3A	General transcription factor IIIA
HAS	Hyaluronan synthase
HLA-DR	Human leukocyte antigen-DR
ICRS	International Cartilage Repair Score
IHC	Immunohistochemistry
IPFP	Infrapatellar fat pad
ISCT	International Society for Cell and Gene Therapy
KL	Kellgren–Lawrence
LPL	Lipoprotein lipase
M	Male
MeSH	Medical Subject Headings
MFI	Median fluorescence intensity
mRNA	Messenger RNA
MSC	Mesenchymal stem/stromal cell
N/A	Not applicable
NGFR	Nerve-growth-factor receptor
NS	Not statistically significant
OA	Osteoarthritis
OHAT	Office of Health Assessment and Translation
OLT	Osteochondral lesion of the talus
P	Passage
PBS	Phosphate-buffered saline
PCNA	Proliferating-cell nuclear antigen
PDGF	Platelet-derived growth factor
PDGFR	Platelet-derived growth-factor receptor
PPAR-γ	Peroxisome proliferator-activated receptor γ
PRG4	Proteoglycan 4
PRISMA	Preferred Reporting Items for Systematic Reviews and Meta-analyses
PROSPERO	International Prospective Register of Systematic Reviews
qPCR	Quantitative polymerase chain reaction
RUNX2	Runt-related transcription factor 2
SCF	Subcutaneous fat
SF	Synovial fluid
SM	Synovial membrane
SOX	SRY-box transcription factor (e.g., SOX6, SOX9)
STRO-1	Stromal precursor antigen-1
TKA	Total knee arthroplasty
VCAM-1	Vascular cell-adhesion molecule 1
VEGFR	Vascular endothelial growth-factor receptor
vK	von Kossa
α-SMA	α-smooth muscle actin
